# Glory of piezoelectric perovskites

**DOI:** 10.1088/1468-6996/16/4/046001

**Published:** 2015-08-03

**Authors:** Kenji Uchino

**Affiliations:** International Center for Actuators & Transducers, The Pennsylvania State University, University Park, PA 16802, USA

**Keywords:** piezoelectric perovskite, electrostriction, relaxor-PT single crystal, photostriction, magnetoelectric composite, multi-functional effect, Pb-free piezoelectrics

## Abstract

This article reviews the history of piezoelectric perovskites and forecasts future development trends, including Uchino’s discoveries such as the Pb(Mg_1/3_Nb_2/3_)O_3_–PbTiO_3_ electrostrictor, Pb(Zn_1/3_Nb_2/3_)O_3_–PbTiO_3_ single crystal, (Pb, La)(Zr, Ti)O_3_ photostriction, and Pb(Zr, Ti)O_3_–Terfenol magnetoelectric composites. We discuss five key trends in the development of piezomaterials: performance to reliability, hard to soft, macro to nano, homo to hetero, and single to multi-functional.

## 1. Introduction

Perovskite ceramics are current primary piezoelectric materials widely commercialized and applied to various devices such as sensors and actuators. This paper reviews the history of piezoelectric perovskites and forecasts the future development directions from the author’s viewpoint.

**Figure 1.**
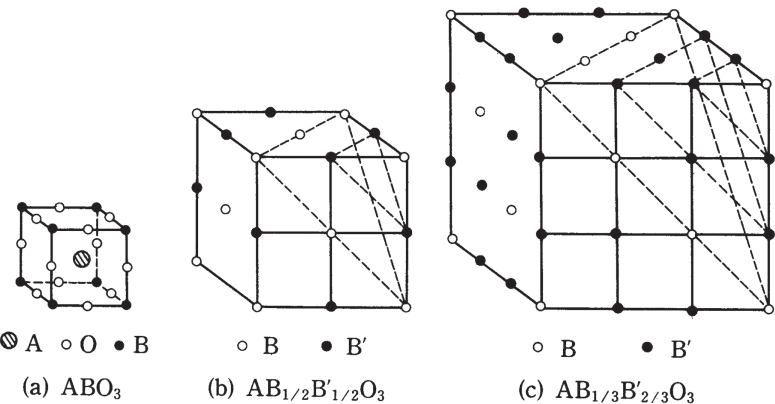
B-ion ordered arrangements in complex perovskites: (a) simple structure, (b) 1:1 ordering, and (c) 1:2 ordering [1]. Solid solution systems can be easily synthesized for tuning the physical properties.

Let us consider first simple and complex perovskites, BaTiO_3_, Pb(Mg_1/3_Nb_2/3_)O_3_, Pb(Mg_1/2_W_1/2_)O_3_, and Ba(Mg_1/3_Ta_2/3_)O_3_, as examples, from a crystallographic viewpoint. Figure 1 illustrates B-ion ordered arrangements in complex perovskites [1]. BaTiO_3_ shows a simple perovskite structure (figure 1(a)). Because Mg^2+^ and Nb^5+^ are arranged randomly (disordered structure) in Pb(Mg_1/3_Nb_2/3_)O_3_, it also shows a simple structure. In contrast, in Pb(Mg_1/2_W_1/2_)O_3_, Mg^2+^ and W^6+^ are arranged in a NaCl type, which is called ‘1:1 ordering’ (figure 1(b)). Ba(Mg_1/3_Ta_2/3_)O_3_ also shows an ordered structure, as illustrated in figure 1(c), where Mg^2+^ and Ta^5+^ are arranged like Mg^2+^–Ta^5+^–Ta^5+^– ···· (called ‘1:2 ordering’). With increasing the ionic valence difference, these ions increase in short-range ordering tendency. Because the ionic ordering provides a significant difference in physical properties, in general, and the solid solution systems between these complex perovskites can be synthesized rather easily, perovskite materials have been studied comprehensively from practical application viewpoints.

This article reviews the history of piezoelectric perovskites and forecasts the future developments. We will discuss five key trends: performance to reliability, hard to soft, macro to nano, homo to hetero, and single to multi-functional.

## 2. Historical background

This section contains a brief summary of piezoelectric perovskites based on [2].

### 2.1. The dawn of piezoelectrics

The Curie brothers (Pierre and Jacques Curie) discovered the direct piezoelectric effect in single crystal quartz in 1880. Under pressure, quartz generated electrical charge/voltage.The root of the word ‘piezo’ means ‘pressure’ in Greek; hence, the original meaning of the word ‘piezoelectricity’ implied pressure-electricity. Materials showing this phenomenon also conversely exhibit a geometric strain/deformation proportional to an applied electric field. This is the ‘converse piezoelectricity’, discovered by Gabriel Lippmann in 1881.

On 15 April 1912, the ship Titanic sank after colliding with an iceberg. This tragic incident could be prevented if ultrasonic sonar had been developed. It motivated the development of ultrasonic technology using piezoelectricity, more than 30 years after its discovery.

### 2.2. World War I: underwater acoustic devices

World War I in 1914 created real demand to fund ultrasonic technology in order to detect German U-boats underwater. This is the strongest force both from social and political demands. Paul Langevin, a professor of Ecole Supérieure de Physique et de Chimie Industrielles de la Ville de Paris (ESPCI Paris Tech) started the experiment on ultrasonic signal transmission in seawater in collaboration with the French Navy. Langevin succeeded in transmitting an ultrasonic pulse in southern France in 1917. A high frequency (40 kHz) was chosen at that time for the sound wave frequency for better monitoring resolution of the objective (U-boats); however, it also led to a rapid decrease in the reachable distance. Note that quartz and Rochelle salt single crystals were the only available piezoelectric materials in the early 20th century. Since the sound velocity in quartz is about 5 km s^−1^, 40 kHz corresponds to the wavelength of 12.5 cm in quartz. In those years, it was not possible to produce high quality quartz single crystals of that size [3].

In order to overcome this dilemma, Langevin invented a new transducer construction; small quartz crystals arranged in a mosaic were sandwiched by two steel plates, which are called the ‘Langevin type’. Though the mechanical quality factor is significantly high (i.e., low loss) in quartz, the major problem for this transducer’s application is its low electromechanical coupling *k*, resulting in (1) low mechanical underwater transmitting power and receiving capability, and in (2) narrow frequency bandwidth, in addition to the practical fact that only Brazil produced natural quartz crystals at that time. Thus, US researchers used Rochelle salt single crystals, which had a superior electromechanical coupling factor (*k* close to 100% at 24 °C) and were easy to grow. Nicholson [4], Anderson, and Cady undertook research on piezoelectric underwater transducers during World War I.

Rochelle salt is sodium potassium tartrate [NaKC_4_H_4_O_6_ · 4H_2_O] and has two Curie temperatures at −18 °C and 24 °C with a very narrow operating temperature range for exhibiting ferroelectricity, leading to high electromechanical coupling at 24 °C [5]. However, its performance has a rather large temperature dependence. It was used worldwide for underwater transducer applications until barium titanate and PZT were discovered. Since this crystal is water-soluble, it will inevitably be degraded by humidity. However, the most delicate problem is its weakness to dryness, but no researcher has invented the necessary coating technology for Rochelle salt devices to achieve the required lifetime.

### 2.3. World War II: discovery of barium titanate

**Figure 2.**
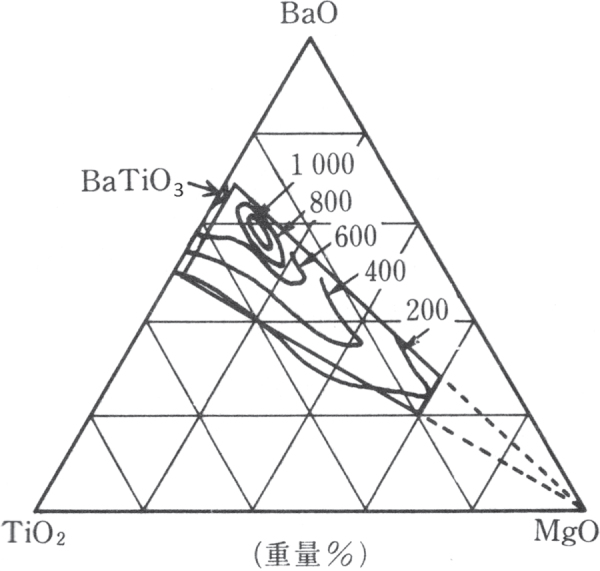
Permittivity contour map on the MgO-TiO_2_-BaO system and the patent coverage composition range (dashed line) [9]. Original Japanese article is used intentionally.

Barium titanate (BaTiO_3_, BT) ceramics were discovered during World War II independently in three countries: the US, by Wainer and Salomon [6] in 1942, Japan by Ogawa [7] in 1944, and Russia by Vul [8] in 1944. This is the dawn of the ‘glory of piezoelectric perovskites’. Compact radar system development requested compact high capacitance ‘condensers’ (the terminology ‘condenser’ was used at that time, rather than ‘capacitor’). Based on the widely used ‘Tita-Con’ (titania condenser) composed of TiO_2_–MgO, researchers doped various ternary oxides to find higher permittivity materials. According to the memorial article authored by Ogawa and Waku [9], they investigated three dopants, CaO, SrO, and BaO, in a wide fraction range. They found a maximum permittivity around the compositions CaTiO_3_, SrTiO_3_, and BaTiO_3_ (all were identified as a *perovskite* structure). In particular, permittivity higher than 1000 in BaTiO_3_ was enormous (10 times higher than Tita-Con) at that time, as illustrated in figure 2. It should be pointed out that the original discovery of BaTiO_3_ was not related to piezoelectric properties, but to high capacitance. Equally important are the independent discoveries by Gray at Erie Resister (patent applied in 1946) [10] and by Roberts at MIT (published in 1947) [11] that the electrically poled BT exhibited piezoelectricity owing to the domain re-alignment. At that time, researchers were arguing that the randomly oriented polycrystalline sample should not exhibit piezoelectricity, but the secondary effect, electrostriction. From this sense, Gray is the ‘father of piezoceramics’, as he first verified that the polycrystalline BT exhibited piezoelectricity once it was electrically poled.

The easiness in composition selection and manufacturability of BT ceramics prompted Mason [12] and others to study transducer applications with these electroceramics. Piezoelectric BT ceramics had a reasonably high coupling coefficient and non-water solubility, but the bottlenecks were (1) a large temperature coefficient of electromechanical parameters because of the second phase transition (from tetragonal to rhombohedral) around room temperature or operating temperature, and (2) the aging effect due to the low Curie temperature (phase transition from tetragonal to cubic) around only 120 °C. In order to increase the Curie temperature higher than 120 °C, to decrease the second transition temperature below −20 °C, various ion replacements, such as Pb and Ca, were studied. From these trials, a new system, PZT, was discovered.

### 2.4. Discovery of PZT

#### 2.4.1. Pb(Zr, Ti)O_3_ (PZT)

**Figure 3.**
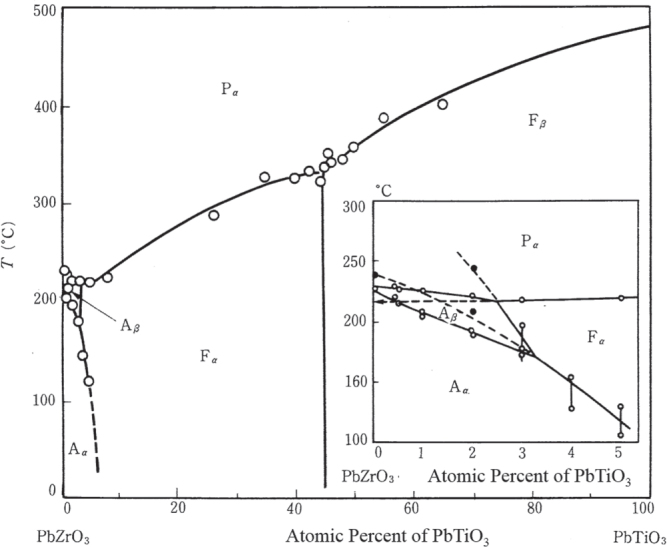
Phase diagram for the Pb(Zr,Ti)O_3_ solid solution system proposed by Sawaguchi. It does not include another ferroelectric phase below F_*α*_ phase, which was discovered later [14].

Following the methodology taken for the BT discovery, perovskite isomorphic oxides such as PbTiO_3_, PbZrO_3_, and SrTiO_3_ and their solid solutions were intensively studied. In particular, the discovery of ‘antiferroelectricity’ in lead zirconate [13] and the determination of the Pb(Zr,Ti)O_3_ (PZT) system phase diagram [14] by Sawaguchi *et al* are noteworthy. Figure 3 shows the original phase diagram of the Pb(Zr,Ti)O_3_ solid solution system reported by Sawaguchi *et al*, which was read and cited worldwide, and triggered the PZT era. We know another ferroelectric phase below F_*α*_ phase nowadays.

A similar discovery history as for barium titanate was repeated for the lead zirconate titanate system. The material was discovered by Sawaguchi *et al*, but the discovery of its superior piezoelectricity was conducted by Jaffe in 1954. Jaffe was at the US National Bureau of Standards at that time. He knew about Sawaguchi *et al*’s serial studies on the PZT system, and focused on the piezoelectric measurement around the so-called *morphotropic phase boundary* (MPB) composition between the tetragonal and rhombohedral phases, and found enormous electromechanical coupling around that composition range [15]. His patent had a significant effect on the future development strategies of Japanese electroceramic industries. Remember two important notions for realizing superior piezoelectricity: (1) Pb-included ceramics, and (2) MPB compositions.

#### 2.4.2. Clevite Corporation

The Brush Development Company manufactured Rochelle salt single crystals and their bimorph components for phonograph applications in the 1930s, and in the 1940s they commercialized piezoelectric quartz crystals by using a hydrothermal process. There was a big piezoelectric group in Brush, led by Hans Jaffe. However, in 1952, the Clevite Corporation was formed by merging the Cleveland Graphite Bronze Co. and Brush, and H Jaffe welcomed B Jaffe from NBS to Clevite and accelerated the PZT business. Their contribution to developing varieties of PZTs (i.e., hard and soft PZTs) by doping acceptor (Mn) and donor (Nb) ions is noteworthy. By the way, PZT was the ‘trademark’ of Clevite, which had not been used by other companies previously, and H Jaffe and B Jaffe were not related at all (just coincidentally had the same last name). These episodes are described in their famous Bible-like book Jaffe, Cook, and Jaffe’s *Piezoelectric Ceramics* [16].

Clevite first concentrated on high quality military and commercial piezoelectric filters. In the mid-1960s, they tried to develop consumer filters for AM radios, especially automobile radios, but did not meet cost initially. However, after 1967, they successfully started mass-production of 10.7 MHz ceramic filters for FM automobile radios, and delivered them to Philco-Ford. Clevite was bought by Gould Inc. in 1969, and resold to Vernitron in 1970. These drastic business actions terminated the promising piezoelectric filter program initiated by Clevite.

#### 2.4.3. Murata Manufacturing Company

The Murata Manufacturing Co., Ltd was founded by Murata in 1944. He learned ceramic technology from his father, Chairman of the former Murata Pottery Manufacturing Co. Murata Manufacturing Company was started with 10 employees to produce electroceramic components. After World War II, under the guidance of Tetsuro Tanaka, a professor at Kyoto University and one of the promoters of the Barium Titanate Study Committee during WWII, Murata started intensive studies on devices based on barium titanate ceramics. The first products with barium titanate ceramics were 50 kHz Langevin-type underwater transducers for fish-finders in Japan [17]. The second products were mechanical filters [18].

In 1960, Murata decided to introduce PZT ceramics by paying a royalty to Clevite Corporation. As already mentioned in the previous section, because of the disappearance of Clevite from the filter business, Murata increased their worldwide share in the ceramic filter products.

#### 2.4.4. Ternary system

Since PZT was protected by the patent of Clevite, USA, subsequently, ternary solid solutions based on PZT with another perovskite phase were investigated intensively by Japanese ceramic companies in the 1960s, thanks to the ease of synthesis of perovskite solid solutions. Examples of these ternary compositions are: PZTs in solid solution with Pb(Mg_1/3_Nb_2/3_)O_3_ (Matsushita-Panasonic), Pb(Zn_1/3_Nb_2/3_)O_3_ (Toshiba), Pb(Mn_1/3_Sb_2/3_)O_3_, Pb(Co_1/3_Nb_2/3_)O_3_, Pb(Mn_1/3_Nb_2/3_)O_3_, Pb(Ni_1/3_Nb_2/3_)O_3_ (NEC), Pb(Sb_1/2_Sn_1/2_)O_3_, Pb(Co_1/2_W_1/2_)O_3_, and Pb(Mg_1/2_W_1/2_)O_3_ (Du Pont), all of which were patented by different companies (almost all composition patents have already expired). The ternary systems with higher material-designing flexibility exhibit, in general, better performance than the binary PZT system, which created advantages for the Japanese manufacturers over Clevite and other US companies.

### 2.5. Lithium niobate/tantalate

Lithium niobate and tantalate have the same chemical formula ABO_3_ as BaTiO_3_ and Pb(Zr,Ti)O_3_. However, the crystal structure is not perovskite, but ilmenite. Ferroelectricity in single crystals of LiNbO_3_ (LN) and LiTaO_3_ (LT) was discovered in 1949 by Matthias and Remeika at Bell Telephone Laboratories [19]. Since the Curie temperatures in these materials are high (1140 °C and 600 °C for LN and LT, respectively), perfect linear characteristics can be observed in electro-optic, piezoelectric, and other effects at room temperature. Though fundamental studies had been conducted, particularly in electro-optic and piezoelectric properties, the commercialization was not accelerated initially because the figure of merit was not very attractive in comparison with perovskite ceramic competitors.

Since Toshiba, Japan started mass-production of LN single crystals after the 1980s, drastic production cost reduction was established. Murata commercialized surface acoustic wave (SAW) filters, which used the SAW mode on LN single crystal. The reader can refer to [20] for recent development of electro-optic light valves, switches, and photorefractive memories, which are improved by optical communication technologies.

### 2.6. Relaxor ferroelectrics: ceramics and single crystals

After the discovery of barium titanate and PZT, in parallel to the PZT-based ternary solid solutions, complex perovskite structure materials were intensively synthesized and investigated in the 1950s. In particular, the contribution by the Russian group led by Smolenskii was enormous. Among them, huge dielectric permittivity was reported in Pb(Mg_1/3_Nb_2/3_)O_3_ (PMN) [21] and Pb(Zn_1/3_Nb_2/3_)O_3_ (PZN) [22]. PMN-based ceramics became major compositions for high-K (∼10 000) capacitors in the 1980s.

**Figure 4.**
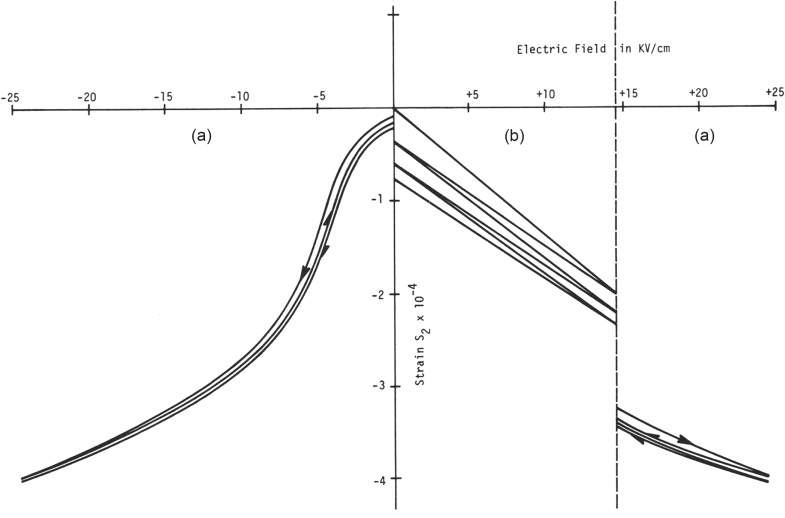
Transverse strain in ceramic specimens of 0.9 PMN-0.1 PT (a) and a typical hard PZT eight piezoceramic (b) under varying electric field cycles [23].

It is noteworthy to introduce two epoch-making discoveries in the late 1970s and early 1980s, relating to electromechanical couplings in relaxor ferroelectrics: electrostrictive actuator materials, and high-*k* (95%) piezoelectric single crystals. Cross *et al* reported extraordinarily large secondary electromechanical coupling, i.e., an ‘electrostrictive’ effect, with the strain level higher than 0.1% at room temperature, exhibiting negligible hysteresis during rising and falling electric field, in a composition 0.9 PMN—0.1 PbTiO_3_ (see figure 4) [23]. Every phenomenon has primary and secondary effects, which are sometimes recognized as linear and quadratic phenomena, respectively. In actuator materials, these correspond to the ‘piezoelectric’ and ‘electrostrictive’ effects.

When the author started actuator research in the middle of the 1970s, precise ‘displacement transducers’ (we initially used this terminology) were required in the Space Shuttle program, particularly for ‘deformable mirrors,’ for controlling the optical pathlengths over several wavelengths (1 *μ*m). Conventional piezoelectric PZT ceramics were plagued by hysteresis and aging effects under large electric fields; this was a serious problem for an optical positioner. Electrostriction, which is the secondary electromechanical coupling (parabolic strain curve as in figure 4) observed in centro-symmetric crystals, is not affected by hysteresis or aging [20]. In addition, the electric poling process is not required. However, at that time, most of the people believed that the secondary effect would be minor, and could not provide a larger contribution than the primary effect. Of course, this may be true in most cases, but the author’s group actually discovered that relaxor ferroelectrics, such as lead magnesium niobate-based solid solutions, exhibit enormous electrostriction. This discovery, in conjunction with Uchino’s multilayer actuator invention (co-fired technique) (1978) with the NEC Corporation, accelerated the development of piezoelectric actuators after the 1980s.

**Figure 5.**
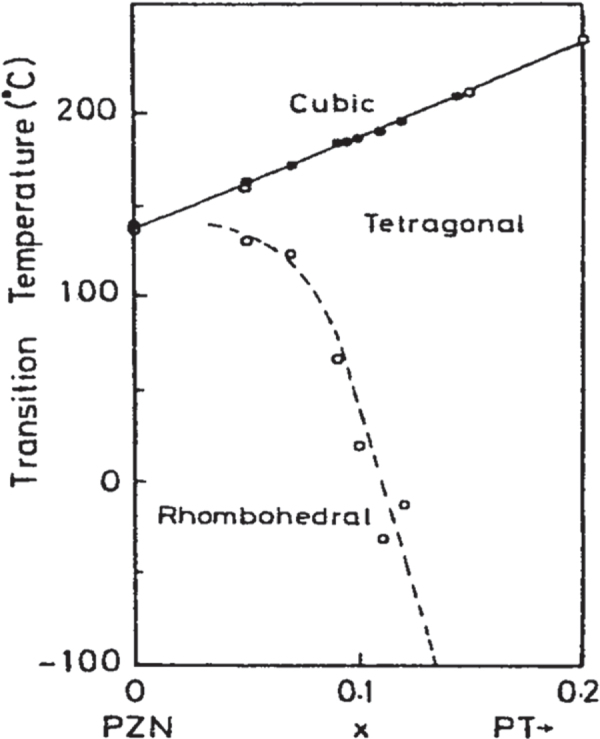
Phase diagram for the Pb(Zn_1/3_Nb_2/3_)O_3_-PbTiO_3_ solid solution system [24].

**Figure 6.**
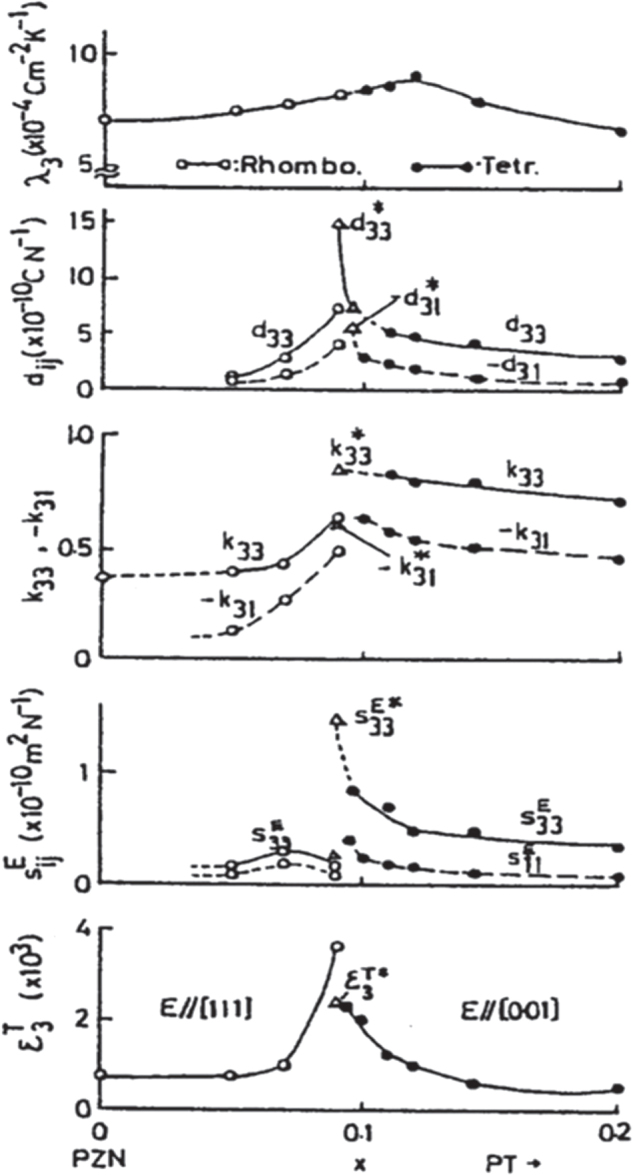
Changes in electromechanical coupling factors with mole faction of PT in the (1−*x*)Pb(Zn_1/3_Nb_2/3_)O_3_-*x*PbTiO_3_ solid solution system [24].

Nomura and Uchino’s group was interested in making single crystals of PZT in the 1970s, in order to clarify the crystal orientation dependence of the piezoelectricity. However, it was difficult to prepare large single crystals around the MPB compositions (52/48). Thus, we focused on the Pb(Zn_1/3_Nb_2/3_)O_3_-PbTiO_3_ solid solution system, which has a phase diagram similar to the PZT system, but large single crystals are rather easily prepared. Refer to the MPB between the rhombohedral and tetragonal phases in figure 5, in comparison with figure 3 for PZT [24]. Figure 6 shows changes in electromechanical coupling factors with mole fraction *x* of PbTiO_3_ (PT) in the (1–*x*) Pb(Zn_1/3_Nb_2/3_)O_3_-*x*PbTiO_3_ solid solution system, reported by Kuwata, Uchino, and Nomura in 1982 [25], which was best cited in 1998 (17 years after the publication). Note that the MPB composition, 0.91 PZN-0.09 PT, exhibited the maximum for all parameters as expected, but the highest values in electromechanical coupling factor *k*_33_∗ and the piezoelectric constant *d*_33_∗ reached 95% and 1600 pC N^−1^. The notation ∗ was used at that time because these values are not normally determined, but for a sample poled along the [100] perovskite direction different from the spontaneous polarization direction 〈111〉, as described below. When a young PhD student, J Kuwata, reported to the author first, even I myself could not believe these large numbers. Thus, we worked together to re-examine the experiments. When I saw the antiresonance frequency of almost twice the resonance frequency, I needed to believe the incredibly high-*k* value. The author still remembers that the first submission of our manuscript had been rejected because the referee could not ‘believe this large value’. The maximum *k*_33_ in 1980 was about 72% in PZT-based ceramics. The paper was published after one year communication by sending the raw admittance curves etc. However, our original discovery was not believed or not required for applications until the middle of the 1990s.

High-*k* piezoelectric materials have been studied for applications in medical acoustics since the mid-1990s. Toshiba started reinvestigation of PZN-PT single crystals with a strong crystal manufacturing background of lithium niobate in the 1980s. The data reported 15 years prior have been reconfirmed, and improved data were obtained, aiming at medical acoustic applications [26]. In parallel, Park and Shrout at Penn State University demonstrated strains as large as 1.7% induced practically for the PZN-PT solid solution single crystals [27]. The reader may know the present application fever of these single crystals, sponsored by the US Navy. The single crystal relaxor ferroelectric is one of the rare examples, which was revived 15 years after its original discovery.

**Figure 7.**
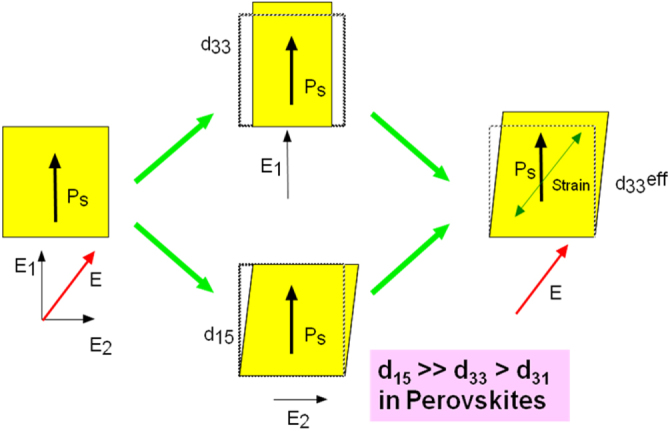
Schematic of the piezoelectricity enhancement depending on the crystal orientation in perovskite ferroelectrics [28]. The large *d*_15_ is the key.

It is notable that the highest values are observed for a rhombohedral composition only when the single crystal is poled along the perovskite [001] axis, not along the [111] spontaneous polarization axis. Figure 7 illustrates an intuitive principle model in understanding this piezoelectricity enhancement depending on the crystal orientation in perovskite ferroelectrics. The key is the largest electromechanical coupling for the *d*_15_ *shear mode* in perovskite structures, because of easy rotation of the oxygen octahedron, in comparison with the squeeze deformation of the octahedron. The reader can refer to the theoretical paper by Du, Belegundu, and Uchino [28]. The concept of the current ‘domain engineering’ is almost analogous to the octahedron rotation.

### 2.7. Composites

Although PZT is superior, due to the high density and acoustic impedance mismatch with water, flexible and lightweight transducers were highly required for underwater and medical acoustic monitoring. Thus, the PZT composite developments started from the end of the 1970s.

#### 2.7.1. Composite effects

**Figure 8.**
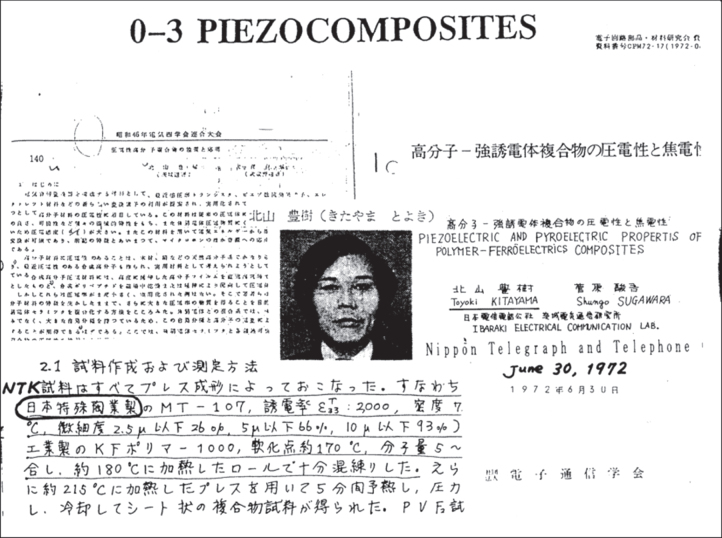
The first report on piezoelectric composites by Kitayama in 1972 [29].

In 1972, Kitayama and Sugawara from Nippon Telegraph and Telephone reported on piezoceramic/polymer composites at Japan IEEE Conference, which seems to be the first paper of the piezoelectric-based composites [29]. As shown in figure 8, their paper dealt with hot-rolled composites made from PZT powder and polyvinylidene difluoride (PVDF), one of the piezoelectric polymer materials, and reported on the piezoelectric and pyroelectric characteristics. Similar flexibility but better piezoelectric performance than PVDF was obtained.

**Figure 9.**
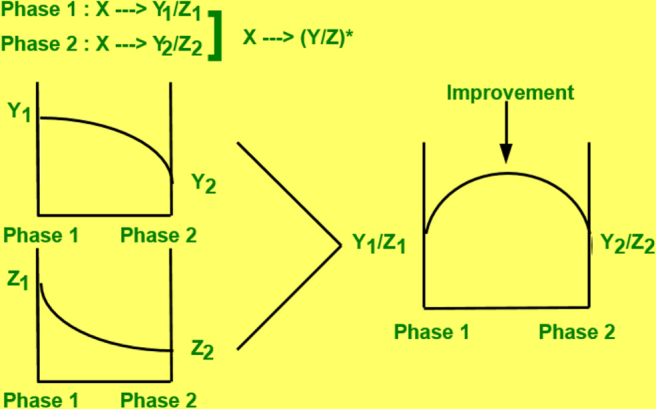
Basic concept of the performance improvement in a composite via a combination effect [30].

Robert E Newnham’s contribution to establishing the *composite connectivity* concept, and the summary of sum, combination, and product effects promoted systematic studies in the piezo-composite area [30]. In certain cases, the averaged value of the output of a composite does exceed both outputs of phase 1 and phase 2. Let us consider two different outputs, Y and Z, for two phases (i.e., Y_1_, Z_1_; Y_2_, Z_2_). When a figure of merit (FOM) for an effect is provided by the fraction (Y/Z), we may expect an extraordinary effect. Suppose that Y and Z follow the convex and concave type sum effects, respectively, as illustrated in figure 9, the combination value Y/Z will exhibit a maximum at an intermediate ratio of phases; that is, the average FOM is higher than either end member FOMs (Y_1_/Z_1_ or Y_2_/Z_2_). This was named the ‘*combination effect*’. Newnham’s group studied various connectivity piezoceramic/polymer composites, which exhibited a combination property of *g* (the piezoelectric voltage constant) which is provided by *d*/*∊*_0_*∊* (*d*: piezoelectric strain constant, and *∊*: relative permittivity), where *d* and *∊* follow the convex and concave type sum effects.

The advantages of the 1–3 composites (PZT rods aligned in polymer matrix) are high coupling factors, low acoustic impedance, good matching to water or human tissue (50–75% of a human body is water!), mechanical flexibility, broad bandwidth in combination with a low mechanical quality factor and the possibility of making undiced arrays by simply patterning the electrodes. The thickness-mode electromechanical coupling of the composite can exceed the *k*_*t*_ (0.40 ∼ 0.50) of the constituent ceramic, approaching almost the value of the rod-mode electromechanical coupling, *k*_33_ (0.70 ∼ 0.80) of that ceramic [31]. The acoustic match to tissue or water (1.5 Mrayls) of the typical piezoceramics (20 ∼ 30 Mrayls) is significantly improved when they are incorporated in a composite structure, that is, when a dense, stiff ceramic is partly replaced with a light, soft polymer. Piezoelectric composite materials are especially useful for underwater sonar and medical diagnostic ultrasonic transducer applications.

#### 2.7.2. Piezoelectric dampers

An intriguing application of PZT composites is a passive mechanical damper. Consider a piezoelectric material attached to an object such as an automobile engine whose vibration is to be damped. When vibration is transmitted to the piezoelectric material, the vibration energy is converted into electrical energy by the piezoelectric effect, and an ac voltage is generated. If a proper resistor is connected, however, the energy converted into electricity is consumed in Joule heating of the resistor, and the amount of energy converted back into mechanical energy is reduced, so that the vibration can rapidly be damped. Taking the series resistance as *R*, the capacitance of the piezoelectric material as *C*, the vibration frequency as *f*, damping takes place most rapidly when the series resistor is selected in such a manner that the impedance matching condition, *R* = 1/(2*πf C*), is satisfied [32].

**Figure 10.**
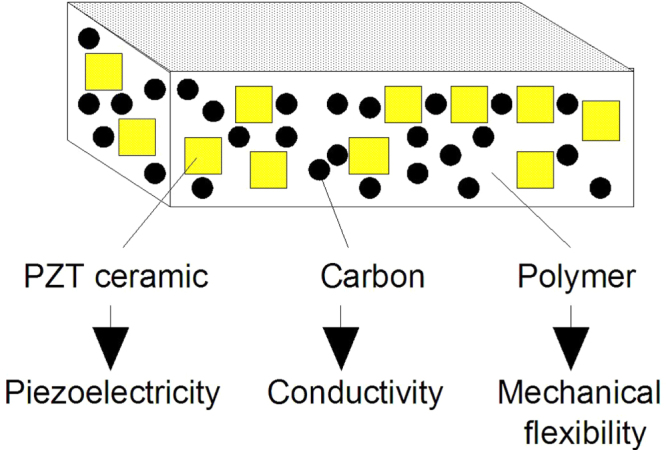
Piezoceramic:polymer:carbon black composite for vibration damping [33].

Being brittle and hard, ceramics are difficult to assemble directly into a mechanical system. Hence, flexible composites can be useful in practice. When a composite of polymer, piezoceramic powder and carbon black is fabricated (figure 10), the electrical conductivity of the composite is greatly changed by addition of small amounts of carbon black (i.e., *percolation effect*) [33]. By properly selecting the electrical conductivity of the composite (i.e., electrical impedance matching), the ceramic powder effectively forms a series circuit with the carbon black, so that the vibration energy is dissipated effectively. The conductivity of the composite changes by more than 10 orders of magnitude around a certain carbon fraction called the ‘*percolation threshold*’, where the carbon powder link starts to be generated. This eliminates the use of external resistors. Note that the damper material exhibits a selective damping performance for a certain vibration frequency, depending on the selected resistivity of the composite, which can be derived from the electrical impedance matching between the permittivity and resistivity.

## 3. Advanced research trends

Base on various resources such as iRAP (Innovative Research & Products), Market Publishers, IDTechEx, our research center summarized that the world 2014 piezoelectric device market size is approximately US$25 billion, with the largest revenue coming from actuator and generator applications, followed by transducers, sensors, accelerators, and piezo-transformers. We can anticipate a 60% growth (USD 40 billion) until 2017.

We will discuss five key piezomaterial development trends in this section for providing the future perspectives of research trends: performance to reliability, hard to soft, macro to nano, homo to hetero, and single to multi-functional. This analysis may be biased toward the author’s personal viewpoint. Part of this section has been published in [34].

### 3.1. Performance to reliability

#### 3.1.1. Pb-free piezoelectrics

In 2006, the European Community started RoHS (Restrictions on the use of certain Hazardous Substances), which explicitly limits the usage of lead (Pb) in electronic equipment. Pb (lead)-free piezoceramics started to be developed after 1999, and are classified into three groups; (Bi, Na)TiO_3_ (BNT), (Na, K)NbO_3_ (NKN) and tungsten bronze (TB), most of which are revival materials after the 1970s.

##### 3.1.1.1. BNT

The share of the patents for bismuth compounds (bismuth layered type and perovskite (Bi, Na)TiO_3_ type) exceeds 61%. This is because bismuth compounds are easily fabricated in comparison with other compounds. Honda Electronics, Japan developed Langevin transducers using BNT based ceramics for ultrasonic cleaner applications [35]. Their composition 0.82(Bi_1/2_Na_1/2_)TiO_3_–0.15BaTiO_3_–0.03(Bi_1/2_Na_1/2_)(Mn_1/3_Nb_2/3_)O_3_ exhibits *d*_33_ = 110 × 10^−12^ C N^−1^, which is only 1/3 of that of a hard PZT, but the electromechanical coupling factor *k*_*t*_ = 0.41 is larger because of the much smaller permittivity (*∊* = 500) than that of PZT. Furthermore, the maximum vibration velocity of a rectangular plate (*k*_31_ mode) is close to 1 m s^–1^ (rms value), which is higher than that of hard PZTs.

##### 3.1.1.2. NKN

**Figure 11.**
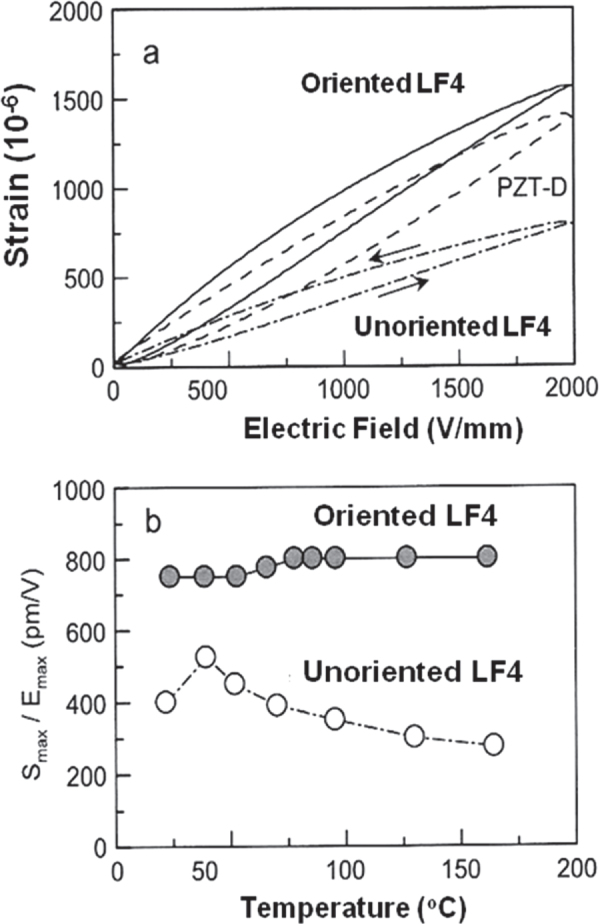
Strain curves for oriented and unoriented (K,Na,Li)(Nb,Ta,Sb)O_3_ ceramics [36].

(Na, K)NbO_3_ systems exhibit the highest performance among the present Pb-free materials, because of the MPB usage. Figure 11 shows the current best data reported by Toyota Central Research Lab, where strain curves for oriented and unoriented (K,Na,Li) (Nb,Ta,Sb)O_3_ ceramics are shown [36]. Note that the maximum strain reaches up to 1500 × 10^−6^, which is equivalent to the PZT strain. Drawbacks include their sintering difficulty and the necessity of the sophisticated preparation technique (topochemical method for preparing flaky raw powder).

##### 3.1.1.3. TB

TB types are another alternative for resonance applications, because of their high Curie temperature and low loss. Taking into account general consumer attitude on disposability of portable equipment, Taiyo Yuden, Japan developed micro-ultrasonic motors using non-Pb multilayer piezo-actuators [37]. Their composition is based on TB ((Sr,Ca)_2_NaNb_5_O_15_) without heavy metal. The basic piezoelectric parameters in TB (*d*_33_ = 55 ∼ 80 pC N^−1^, *T*_*C*_ = 300 °C) are not very attractive. However, once the *c*-axis oriented ceramics are prepared, the *d*_33_ is dramatically enhanced up to 240 pC N^−1^. Further since Young’s modulus 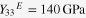 is more than twice of that of PZT, the higher generative stress is expected, which is suitable for ultrasonic motor applications. Taiyo Yuden developed a sophisticated preparation technology for oriented ceramics with a multilayer configuration; that is, preparation under strong magnetic field, much simpler than the flaky powder preparation.

#### 3.1.2. Low loss piezoelectrics

High power piezoelectrics with low loss have become a central research topic from the energy efficiency improvement viewpoint; that is to say, ‘real (strain magnitude) to imaginary performance (heat generation reduction)’. Reducing hysteresis and increasing the mechanical quality factor to amplify the resonance displacement is the primary target from the transducer application viewpoint.

##### 3.1.2.1. Loss characterization principle

We proposed a universal loss characterization methodology in smart materials, piezoelectrics, and magnetostrictors; namely, by measuring accurately the mechanical quality factors *Q*_*A*_ for the resonance and *Q*_*B*_ for the antiresonance in the admittance/impedance curve, we can derive physical losses [38, 39].

There are three losses in piezoelectrics: dielectric tan *δ*, elastic tan *ϕ*, and piezoelectric tan *θ*, each of which is further categorized intensive (stress-free, short-circuited boundary condition) and extensive (strain-free, open-circuited) losses as defined by:

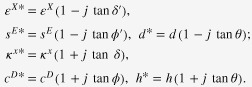


Though the previous researchers neglected the piezoelectric loss (tan *θ*), we pointed out that piezoelectric loss has almost a comparable magnitude with the dielectric and elastic losses, and is essential to explain the admittance/impedance spectrum.

A universal method for determining the piezoelectric loss is summarized for a piezoelectric sample here (e.g., k_31_ mode).

(1) Obtain tan *δ*′ from an impedance analyzer or a capacitance meter at a frequency away from the resonance or antiresonance range.

Obtain the following parameters experimentally from an admittance/impedance spectrum around the resonance (*A*-type) and antiresonance (*B*-type) range: *ω*_*a*_, *ω*_*b*_, *Q*_*A*_, *Q*_*B*_ (from the 3 dB bandwidth method), and the normalized frequency *Ω*_*b*_ = *ω*_*b*_*l*/2*v*.

(2) Obtain tan *ϕ*′ from the inverse value of *Q*_*A*_ (quality factor at the resonance) in the *k*_31_ mode.
(3) Calculate electromechanical coupling factor *k* from the *ω*_*a*_ and *ω*_*b*_ with the IEEE Standard equation in the *k*_31_ mode:

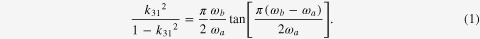
(4) Finally obtain tan *θ*′ by the following equation in the k_31_ mode:

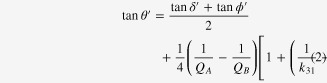


##### 3.1.2.2. HiPoCS

**Figure 12.**
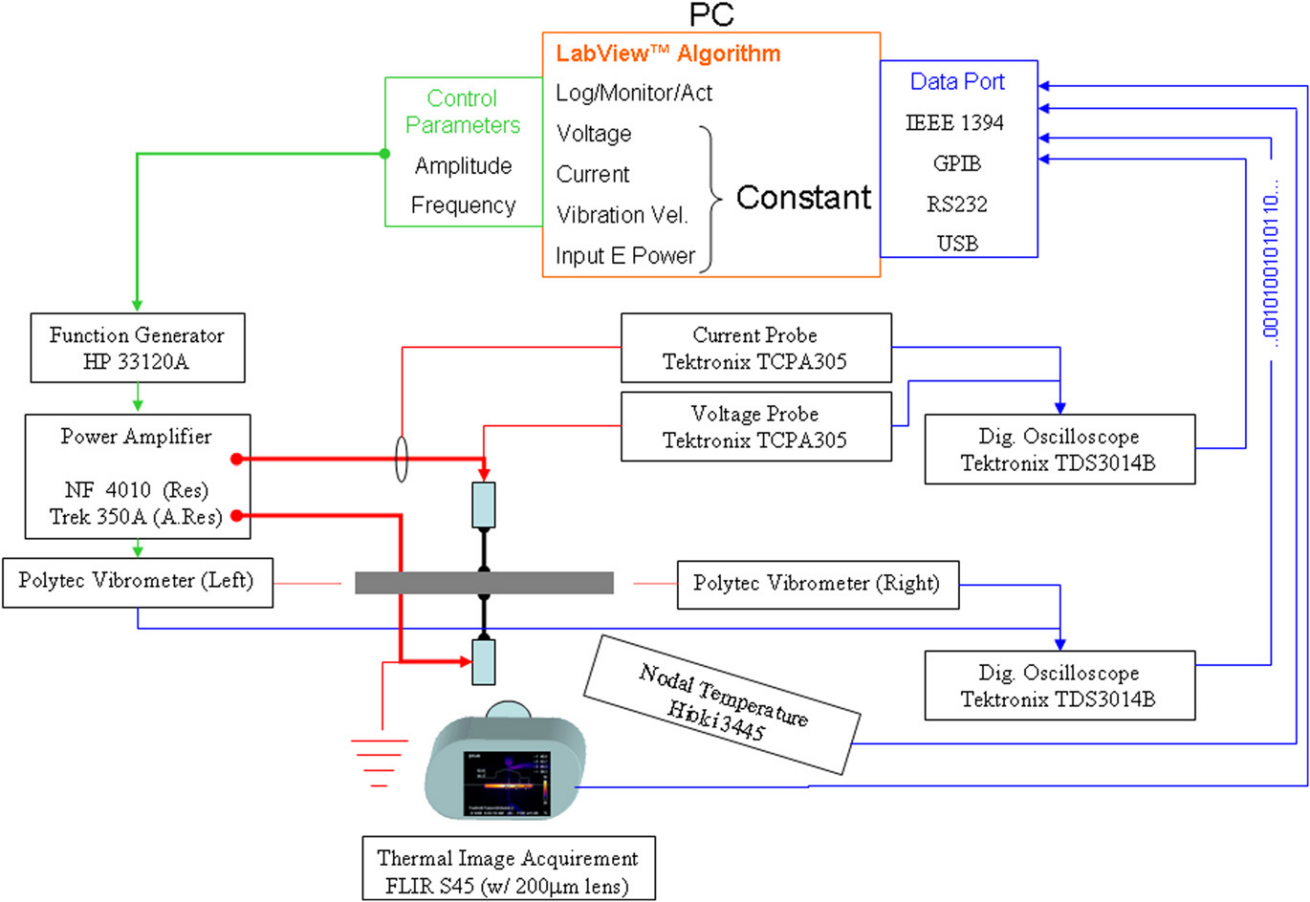
High power piezoelectric characterization system (HiPoCS) developed by ICAT, Penn State University [38].

**Figure 13.**
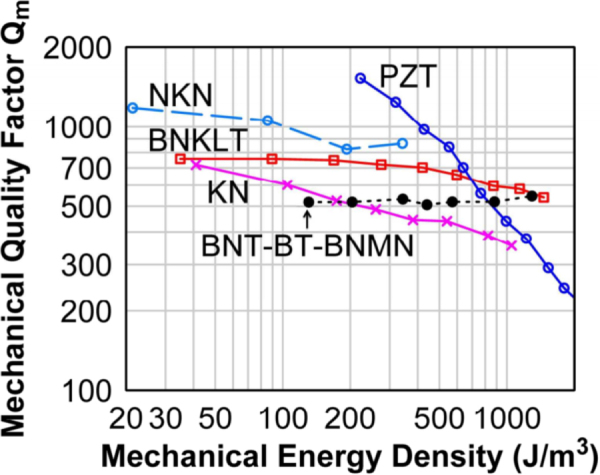
High power characterization of Pb-free piezoelectrics and PZT. Mechanical energy density is higher in some Pb-free ceramics than in PZT.

Figure 12 shows the high power piezoelectric characterization system (HiPoCS™) developed by ICAT at Penn State University [40]. This system can measure the admittance spectra under various conditions (constant voltage, current, vibration velocity, input electric power) or the transient vibration response under pulse drive, which can precisely determine both *Q*_*A*_ and *Q*_*B*_ simultaneously, by escaping from the impedance spectrum distortion, jump, or hysteresis. High power characterization of Pb-free piezoelectric and PZT disk samples is demonstrated in figure 13, where the resonance *Q*_*A*_ is plotted as a function of vibration mechanical energy density 
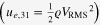
.

**Figure 14.**
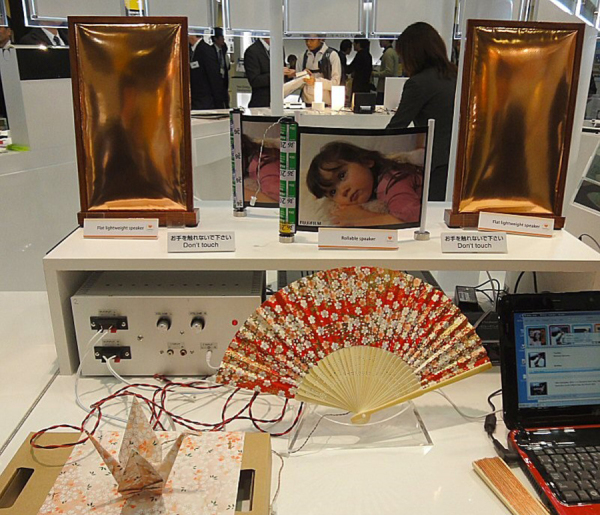
Bendable, foldable speaker [41]. Even a ‘paper crane’ (front) can act as a speaker, which can generate beautiful sound. [Picture: courtesy of Fuji Film.]

Compared with the maximum vibration velocity (defined by the velocity which generates a 20 °C temperature rise on the sample) of 0.3 m s^–1^ (rms) in hard PZT, Pb-free piezoelectrics can exhibit the maximum vibration velocity higher than 0.5 m s^−1^, leading to higher energy density as a transducer (though the mass density *ρ* is smaller).

### 3.2. Hard to soft

Since the 1980s, we face the era of polymers, owing to their superior elasticity. Larger, thinner, lighter, and mechanically flexible human interfaces are the current necessity in portable electronic devices, leading to the development in elastically soft displays, electronic circuits, and speakers/microphones.

#### 3.2.1. 0:3 PZT composites

In addition to electro-active elastomer and PVDF type polymer piezoelectrics, Fuji Film news-released their new bendable and foldable speakers with using a 0:3 composite (PZT fine powder is mixed in a polymer film), as shown in figure 14 [41]. Superior acoustic performance seems to be promising for flat-speaker applications.

#### 3.2.2. Large strain ceramics

As we already introduced, Pb(Zn_1/3_Nb_2/3_)O_3_–PbTiO_3_ (PZN-PT) or Pb(Mg_1/3_Nb_2/3_)O_3_–PbTiO_3_ (PMN-PT) single crystals became focused owing to their rubber-like soft piezoceramic strain after the discovery. Since the enhancement of the induced strain level is a primary target, single crystals with a better capability for generating larger strains are being used these days. Strains as large as 1.7% can be induced practically for the PZN-PT solid solution single crystals [27]. Note again that the highest values are observed for a rhombohedral composition only when the single crystal is poled along the perovskite [001] axis, not along the [111] spontaneous polarization axis. This has accelerated research in ‘domain engineering’ in piezoelectric perovskites [42].

### 3.3. Macro to nano

In the micro-(nano) electromechanical system (MEMS/ NEMS) area, *piezoelectric MEMS* is one of the miniaturization targets for integrating piezo-actuators in a micro-scale device, aiming at bio/medical applications for maintaining human health.

**Figure 15.**
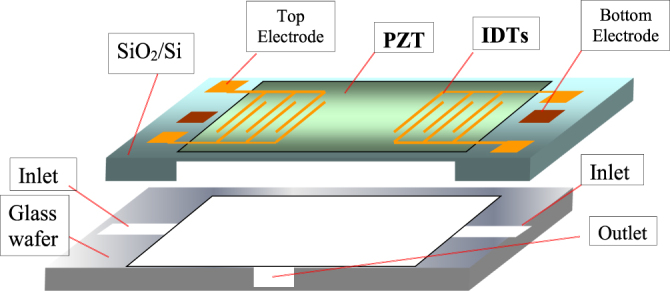
Structure of a PZT/silicon MEMS device, a blood tester [43].

PZT thin films are deposited on a silicon wafer, which is then micro-machined to leave a membrane for fabricating micro-actuators and sensors, i.e., micro-electromechanical systems. Figure 15 illustrates a blood tester developed by Penn State in collaboration with OMRON Corporation, Japan [43]. Applying voltage to two surface interdigital electrodes, the surface PZT film generates surface membrane waves that soak up blood and the test chemical from the two inlets, then mix them in the center part, and send the mixture to the monitor part through the outlet. A finite element analysis calculation was conducted to evaluate the flow rate of the liquid by changing the thickness of the PZT or the Si membrane, inlet and outlet nozzle size, cavity thickness. Refer to [44] for updated piezoelectric MEMS studies.

**Figure 16.**
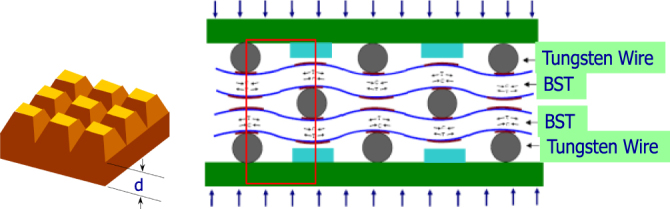
Spatial gradient of stress can be obtained in trapezoidal BST samples or wire-inserted BST laminates.

### 3.4. Homo to hetero

The homo to hetero structure change is also a recent research trend: the stress-gradient in terms of space in a dielectric material exhibits piezoelectric-equivalent sensing capability (i.e., *flexoelectricity*), while the electric-field gradient in terms of space in a semiconductive piezoelectric can exhibit bimorph-equivalent flextensional deformation (*monomorph*).

The spatial gradient of stress or electric field generates direct or converse flexoelectric effect, expressed respectively by:

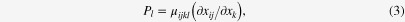


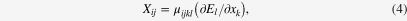
where *P*_*l*_*, Е*_*l*_ are electric polarization, electric field; *X*_*ij*_, *x*_*ij*_ are elastic stress, strain; *x*_*k*_ is coordination in *x*_*ij*_ or *E*_*l*_; *μ*_*ijkl*_ is denoted as a flexoelectric coefficient, which has a fourth rank polar tensor symmetry, similar to the electrostrictive tensor [45]. This means that even a paraelectric material can generate charge under stress when the strain gradient is generated artificially in the material. Cross demonstrated this piezoelectric-equivalent effect in various artificial designs as shown in figure 16 [46]. Ba_0.67_Sr_0.33_TiO_3_ (BST) paraelectric composition sample with a trapezoid shape exhibited 10^−7^ C m^−2^ of polarization under a strain gradient of 10^−3^ m^−1^.

Conventional bimorph bending actuators are composed of two piezoelectric plates, or two plates and an elastic shim, bonded together. The bonding layer in the latter, however, causes both an increase in hysteresis and a degradation of the displacement characteristics, as well as delamination problems. Furthermore, the fabrication process for such devices, which involves cutting, polishing, electroding, and bonding steps, is rather laborious and costly. Thus, a monolithic bending actuator (*monomorph*) that requires no bonding is a very attractive alternative structure.

**Figure 17.**
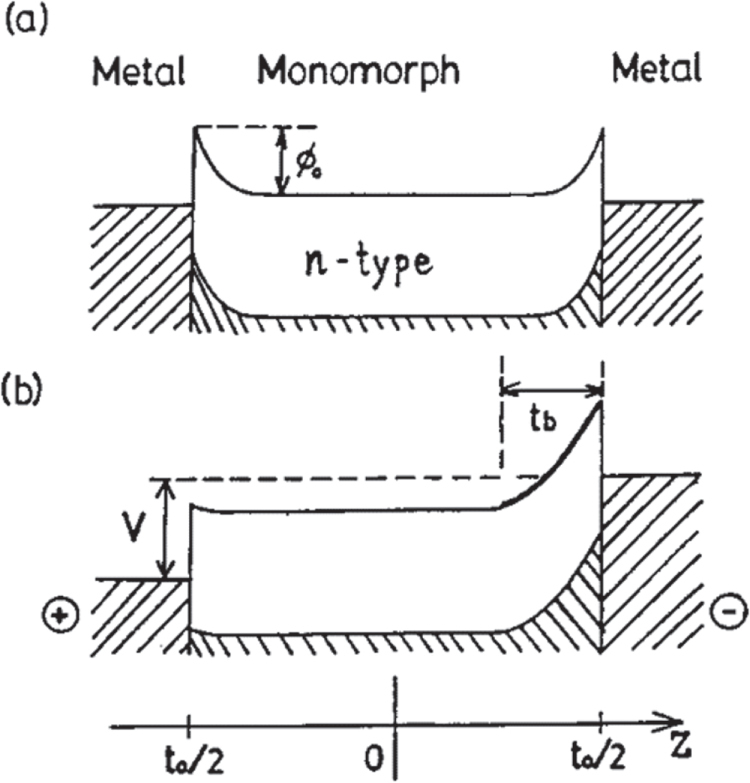
Schottky barrier generated at the interface between a semiconductive (n-type) piezoceramic and metal electrodes [47].

**Figure 18.**
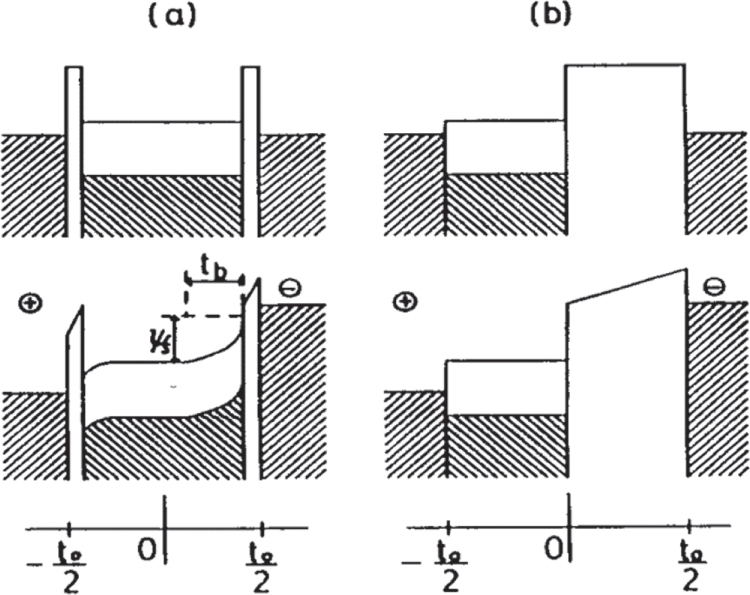
Energy diagrams for modified monomorph structures: (a) a device incorporating a very thin insulating layer and (b) the ‘rainbow’ structure [48].

Such a monomorph device can be produced from a single ceramic plate [47]. The operating principle is based on the combined action of a semiconductor contact phenomenon and the piezoelectric or electrostrictive effect. When metal electrodes are applied to both surfaces of a semiconductor plate and a voltage is applied as shown in figure 17, the electric field is concentrated on one side (that is, *Schottky barrier*), thereby generating a non-uniform field within the uniform plate. When the piezoelectric (or electrostrictor) is slightly semiconductive, contraction along the surface occurs through the piezoelectric effect only on the side where the electric field is concentrated. The non-uniform field distribution generated in the ceramic causes an overall bending of the entire plate. The energy diagram of a modified structure including a very thin insulative layer is represented in figure 18(a) [48]. The thin insulator layer increases the breakdown voltage. The *rainbow actuator* by Aura Ceramics is a modification of the basic semiconductive piezoelectric monomorph design, where half of the piezoelectric plate is reduced to make a thick semiconductive electrode that enhances the bending action [49]. The energy diagram for the rainbow device is shown in figure 18(b) [48].

### 3.5. Single to multi-functional

Some new functions can be realized by coupling two effects. We developed *magnetoelectric* devices (i.e., voltage is generated by applying magnetic field) by laminating magnetostrictive Terfenol-D and piezoelectric PZT materials, and demonstrated *photostriction* by coupling photovoltaic and piezoelectric effects in PLZT.

#### 3.5.1. Magnetoelectric effect

Similar to nuclear radiation, magnetic irradiation cannot be easy felt by humans. We cannot even purchase a magnetic field detector for a low frequency (50 or 60 Hz for power cable).

##### 3.5.1.1. Single-phase approach

Magneto-ferroelectric development was popular in the 1960s and 1970s, which seems to be the initial multi-functional materials boom. Pb(Fe_2/3_W_1/3_)O_3_ was one of the targeted perovskites, which is a relaxor ferroelectric with Curie temperature at 178 K. Because Fe^3+^ and W^6+^ are randomly aligned (disordered perovskite), only the antiferromagnetic phase exists at low temperature. If the ionic arrangement becomes ordered, ferrimagnetism may appear; that is, ferrimagnetic-ferroelectric, which is significantly intriguing from an application viewpoint. From this motivation, Uchino *et al* investigated solid solution systems Pb(Fe_2/3_W_1/3_)O_3_ -Pb(Me_1/2_W_1/2_)O_3_ (Me = Mn, Ni and Co) [50–52]. Pb(Me_1/2_W_1/2_)O_3_ shows antiferroelectric behavior with 1:1 ionic ordering. As expected, the solid solution compositions exhibited 1:1 B-site ordering and ferrimagnetism, and kept their ferroelectric properties. However, because (1) the magnetoelectric coupling (i.e., polarization change with applying magnetic field) is weak, and (2) the performance is observed only at low temperature (<150 K), the application research was not conducted.

##### 3.5.1.2. Composite approach

From the application viewpoint, Philips developed a magnetoelectric material based on the composite product effect concept [53], which exhibits electric voltage under the magnetic field application at room temperature, aiming at magnetic field sensors. This material was composed of magnetostrictive CoFe_2_O_4_ and piezoelectric BaTiO_3_ mixed and sintered together (i.e., 3–3 composite). When a magnetic field is applied on this composite, cobalt ferrite generates magnetostriction, which is transferred to barium titanate as stress, finally leading to the generation of charge/voltage via the piezoelectric effect in BaTiO_3_.

**Figure 19.**
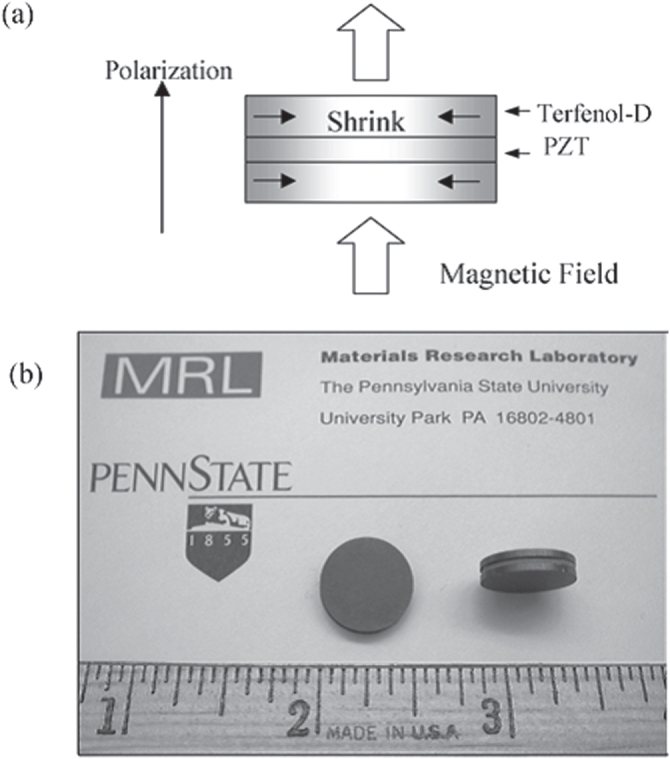
Magnetic noise sensor consisting of a laminated composite of a PZT and two Terfenol-D disks: (a) schematic structure, and (b) photograph of the device [54].

Aiming at further performance improvement, Penn State, in collaboration with Seoul National University, developed a simple and handy magnetic noise sensor for these environmental monitoring purpose, e.g., below a high-voltage power transmission line. Figure 19(a) depicts a schematic structure of this device, in which a PZT disk is sandwiched by two Terfenol-D (magnetostrictor) disks, and a photo of actual sensor device is shown in figure 19(b) [54]. When a magnetic field *H* is applied on this composite, Terfenol-D will expand to be much larger than CoFe_2_O_4_, which is mechanically transferred to PZT (better performance than BT), leading to the higher detection performance (*∂E/∂H*). The key of this device is highly effective for a low frequency such as 50 Hz [54].

#### 3.5.2. Photostriction

The *photostriction* phenomenon was discovered by Dr P S Brody and the present author independently almost at the same time in 1981 [55, 56]. In principle, the photostrictive effect arises from a superposition of the *bulk photovoltaic effect*, i.e., generation of large voltage from the irradiation of light, and the converse-piezoelectric effect, i.e., expansion or contraction under the voltage applied [57]. In certain ferroelectrics, a constant electromotive force is generated with exposure to light, and a photostrictive strain results from the coupling of this bulk photovoltaic effect to converse piezoelectricity. A photostrictive actuator is a fine example of an intelligent material, incorporating illumination sensing and self-production of drive/control voltage together with final actuation.

A bimorph unit has been made from (Pb_0.97_La_0.03_) (Zr_0.52_Ti_0.48_)_0.99_O_3_ (PLZT 3/52/48) ceramic doped with slight addition of tungsten [57]. The remnant polarization of one PLZT layer is parallel to the plate and in the direction opposite to that of the other plate. When a violet light is irradiated to one side of the PLZT bimorph, enormous photovoltage of 1 kV mm^−1^ is generated, causing a bending motion. The tip displacement of a 20 mm long bimorph with 0.4 mm in thickness was 150 *μ*m, with a response time of 1 s.

**Figure 20.**
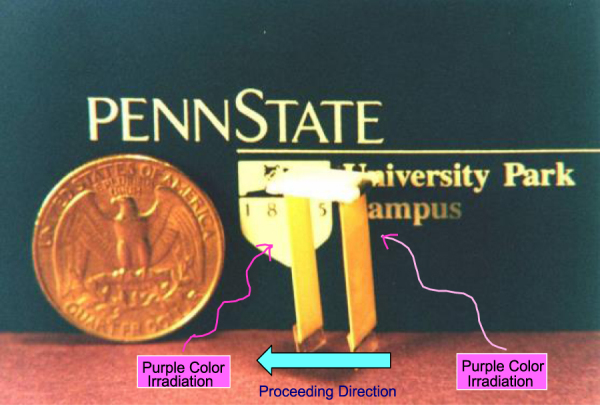
Photo-driven walking device that uses photostrictive PLZT bimorphs [58].

A photo-driven micro-walking device, designed to begin moving by light illumination, has been developed [58]. As shown in figure 20, it is simple in structure, having neither lead wires nor electric circuitry, with two bimorph legs fixed to a plastic board. When the legs are irradiated alternately with light, the device moves like an inchworm with a speed of 100 *μ*m min^−1^. In pursuit of thick film type photostrictive actuators for space structure applications, in collaboration with Jet Propulsion Laboratory, Penn State investigated the optimal range of sample thickness and surface roughness dependence of photostriction. Some 30 *μ*m thick PLZT films exhibit the maximum photovoltaic phenomenon [59].

## 4. Application development trend

In this section, the author emphasizes new trends in sustainability and crisis technologies rather than conventional commercial market developments.

### 4.1. Normal technologies

In the application area, the global regime for ecological sustainability particularly accelerated new developments in ultrasonic disposal technology of hazardous materials, diesel injection valves for air pollution, and piezoelectric renewable energy-harvesting systems.

#### 4.1.1. Ultrasonic disposal technology

Ultrasonic lens cleaner is commonly used in the home. Industrial ultrasonic cleaners are widely utilized in the manufacturing lines of silicon wafers and liquid crystal glass substrates. Honda Electronics added another ultrasonic cleaner in conjunction with a washing machine produced by Sharp Corporation [60]. Using an L–L coupler horn to generate water cavitation, their machine can remove dirt on a shirt collar. It is noteworthy that we can reduce the amount of detergent (one of the major causes of the river contamination) significantly using this technique.

Increasing the power level of water cavitation, we can make hazardous waste innocuous, because the cavitation (cyclic adiabatic compression) generates more than 3000 °C locally for a short period. Hazardous wastes in underground or sewer water include dioxin, trichloroethylene, PCB, and environmental hormones [61]. As is well known, dioxin becomes another toxic material when it is burned at a low temperature, while it becomes innocuous only when burned at a high enough temperature. Ultrasonic cavitation is very useful for this purpose, though the water average temperature does not increase higher than 50 °C.

#### 4.1.2. Reduction of contamination gas

**Figure 21.**
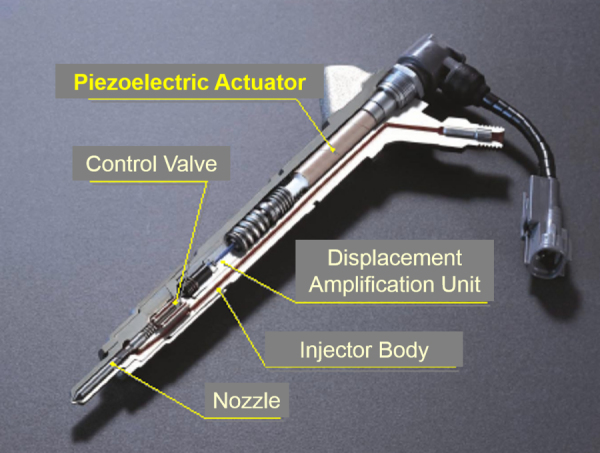
Common rail type diesel injection valve with a piezoelectric multilayer actuator. [Courtesy of Denso Corporation.]

Diesel engines are recommended rather than regular gasoline cars from the energy conservation and global warming viewpoint when we consider the total energy of gasoline production in both well-to-tank and tank-to-wheel processes. The energy efficiency (measured by the total energy required to realize unit drive distance for a vehicle (MJ km^–1^)) is of course better for high-octane gasoline than diesel oil. However, since the electric energy required for purification is significant, gasoline is inferior to diesel [62]. As is well known, the conventional diesel engine, however, generates toxic exhaust gases such as SO_*x*_ and NO_*x*_. In order to solve this problem, new diesel injection valves were developed by Siemens, Bosch, and Toyota with PZT-based piezoelectric multilayered actuators. Figure 21 shows such a common rail type diesel injection valve with a multilayer piezo-actuator that produces high-pressure fuel and quick injection control. The highest reliability of these devices at an elevated temperature (150 °C) for a long period (10 years) has been achieved [63]. The PZT piezoelectric actuator is key to increase burning efficiency and minimize the toxic exhaust gases.

#### 4.1.3. New energy-harvesting systems

**Figure 22.**
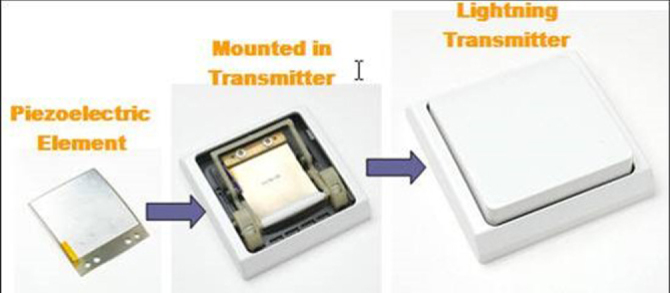
Lightning Switch™ with piezoelectric Thunder™ actuator. [Courtesy of Face Electronics.]

One of the most recent research interests is piezoelectric energy harvesting. Cyclic electric field excited in the piezoelectric plate by the environmental noise vibration is now accumulated into a rechargeable battery without consuming it as Joule heat in original dampers [64, 65]. NEC-Tokin developed an LED traffic light array system driven by a piezoelectric windmill, which is operated by wind generated effectively by passing automobiles. Successful products (million sellers) in the commercial market include the Lightning Switch^TM^ (remote switch for room lights, with using a unimorph piezoelectric component) by PulseSwitch Systems, VA [65]. In addition to the living convenience, Lightning Switch™ (figure 22) can reduce the housing construction cost drastically, due to a significant reduction of the copper electric wire and the aligning labor.

The Penn State group developed energy-harvesting piezoelectric devices based on a ‘Cymbal’ structure (29 mm*ϕ*, 1–2 mm thick), which can generate electric energy up to 100 mW under an automobile engine vibration [66, 67]. This application chased two ‘rabbits’ simultaneously; that is, vibration damping and energy harvesting. By combining three cymbals in a rubber composite, a washer-like energy-harvesting sheet was developed for a hybrid car application, aiming at 1 W-level constant accumulation to a fuel cell.

#### 4.1.4. Medical instruments

Economic recession and the aging phenomenon (average age reached is 87 years old for females in Japan) accelerated medical technologies in advanced countries. As we described in [68], small size, quick response, and high energy density are the strong points of piezoelectric actuators, in comparison with conventional electromagnetic motors. Micro-motors have been tested in medical catheter applications (blood clot removal), and artificial fertilization systems. Large ultrasonic motors can now be found in magnetic resonance imaging (MRI) systems, for escaping from the magnetic interference. Piezo-MEMS devices are targeted to be blood testers, drug delivery systems, etc.

### 4.2. Crisis technology

Crisis technologies are classified into five types:

• natural disasters (earthquakes, tsunamis, tornadoes, hurricanes, lightning, etc),
• epidemic/infectious diseases (smallpox, polio, measles, and HIV),
• catastrophic accident (Three-Mile-Island core meltdown accident, BP oil spill etc),
• intentional damage (terrorism, criminal activity), and
• civil wars, wars, territorial aggression.

#### 4.2.1. Infectious diseases

As infectious or contagious disease involves some association with terrorist activities, those five are related to each other. In the United States, politicians were attacked with anthrax in 2001. In order to neutralize the biological attack, Pezeshk, Gao, and Uchino at Penn State University developed a portable hypochlorous acid disinfection device with using a piezoelectric ultrasonic humidifier [69]. Hypochlorous acid is a strong disinfectant with no side effects for humans and would be ideal for disinfecting office and hospital buildings against deceases like SARS and anthrax. Coupled with the atomization of the acidic solution, much higher disinfection effects can be expected. This acid is not sold as a pure solution since it naturally disintegrates after a few hours. We designed a corrosion-resistant electrolytic cell to produce hypochlorous acid from brine. An ultrasonic piezoelectric atomizer was utilized to generate micro-droplets of the diluted acid.

#### 4.2.2. Natural disasters

Regarding natural disasters, the research themes of urgent need in the actuator/sensor area include: (1) prediction technologies such as for earthquakes, tornadoes, (2) accurate monitoring and surveillance techniques, (3) technologies for gathering and managing crisis information and informing the public in a way not to bring about panic, and (4) rescue technologies (autonomous unmanned underwater, aerial, land vehicles, robots). PMN-PT single crystals are now widely tested for high-resolution sonar systems for surveillance purposes.

## 5. Summary

The history of ferroelectric/piezoelectric perovskites was introduced in the first part of this review. Then, we discussed five key future trends: performance to reliability (Pb-free piezoelectrics, low loss piezo-electrics), hard to soft (foldable piezo-polymer films, PMN/PZN single crystals), macro to nano (piezo-MEMS), homo to hetero (flexo-electricity, mono-morphs), and single to multi-functional (magneto-electrics, photostriction). In the application areas, the global tendency for ecological sustainability accelerated new developments in ultrasonic disposal of hazardous materials, diesel injection valves for air pollution, and piezoelectric renewable energy-harvesting systems. There are also many disaster prevention applications for piezoelectric perovskites, such as earthquake/tsunami monitoring and nuclear power plant safety systems. The author hopes for the ‘glory of piezoelectric perovskites’ in the 21st century.
